# Focal incidental upper abdominal findings on unenhanced chest computed tomography that do not require further imaging: a roadmap for the thoracic radiologist

**DOI:** 10.1590/0100-3984.2021.0106

**Published:** 2022

**Authors:** Henrique Pavan, Tiago Severo Garcia, Felipe Soares Torres, Fernando Ferreira Gazzoni, Luciano Folador, Caroline Lorenzoni Almeida Ghezzi

**Affiliations:** 1 Hospital de Clínicas de Porto Alegre (HCPA), Porto Alegre, RS, Brazil.; 2 Cardiothoracic Division, Department of Medical Imaging, University of Toronto, Toronto, ON, Canada.; 3 Hospital Moinhos de Vento, Porto Alegre, RS, Brazil.

**Keywords:** Incidental findings, Diagnostic imaging, Abdomen/diagnostic imaging, Tomography, X-ray computed, Achados incidentais, Diagnóstico por imagem, Abdome/diagnóstico por imagem, Tomografia computadorizada

## Abstract

Chest scans usually include the upper abdomen, leading radiologists to evaluate the upper abdominal structures. The aim of this article is to summarize the most common incidental upper abdominal findings that do not require further imaging or management in patients undergoing unenhanced computed tomography of the chest for the investigation of thoracic symptoms or diseases. We review common incidental findings of the liver, gallbladder, spleen, adrenal glands, kidney, and retroperitoneum, as well as findings that mimic other lesions. Thoracic radiologists should be aware of such typical findings and report when no further investigation is needed, thus avoiding unnecessary imaging examinations, protecting patients from additional medical interventions, and allaying patient concerns.

## Abstract (pt)

As tomografias de tórax geralmente incluem o abdome superior, o que implica a avaliação das estruturas abdominais superiores. Nosso objetivo é resumir os achados incidentais mais comuns do abdome superior que não requerem imagem ou tratamento adicional em pacientes submetidos a tomografia computadorizada de tórax sem contraste para investigar sintomas ou doenças torácicas. Achados incidentais comuns do fígado, vesícula biliar, baço, glândulas adrenais, rins, retroperitônio e alterações que podem mimetizar lesões nesses órgãos serão revisados. O radiologista torácico deve estar ciente de tais achados e relatar quando nenhuma investigação adicional for necessária, para evitar excesso de exames, especialmente com radiação ionizante, proteger os pacientes de intervenções médicas e de preocupações.

## INTRODUCTION

Most scans of the chest are obtained without contrast enhancement, and the upper abdomen is usually included. The evaluation of the upper abdominal structures is essential, regardless of what the target organ is, and the assessment of the upper abdomen occasionally reveals an abdominal mass or lesion. There are well-established recommendations regarding the management of incidental abdominal findings^(1-5)^. Fortunately, there has been concern about incidental findings, and there are well-established recommendations regarding mediastinal and cardiovascular findings on computed tomography (CT) of the chest^(6)^. Along those same lines, this review aims to aggregate the current recommendations for upper abdominal findings on unenhanced CT that do not require follow-up imaging (Table 1).

**Table 1:** Current recommendations for upper abdominal findings on unenhanced CT that do not require follow-up imaging.

Finding	Imaging features	Comments
Hepatic cyst	Density ranging from -10 to 20 HU	No additional imaging required
Focal fat sparing and focal fatty deposition in the liver	Focal areas of decreased or increased density	No need for immediate invasive evaluation
Gallstones	CT approximately 80% sensitive for the detection of gallstones	In symptomatic patients, ultrasound indicated
Porcelain gallbladder	Focal or diffuse calcification of the gallbladder wall	No evidence supporting imaging follow-up; if followed, use contrast-enhanced CT
Dense gallbladder content	Hyperattenuating gallbladder (20-100 HU)	Clinical history valuable for determining the cause
Splenic cyst	Low attenuation (< 10 HU)	No additional imaging required
Lipid-rich adrenal adenoma	Attenuation ≤ 10 HU	No additional imaging required
Adrenal myelolipoma	Main diagnostic feature: macroscopic fat	No additional imaging required
Likely benign cyst	Well-defined homogeneous mass with density ranging from -9 HU to 20 HU	No additional imaging required
High-attenuation benign cyst	Well-defined homogeneous mass with a density ≥ 70 HU	No additional imaging required
Renal angiomyolipoma	Density of ≤ -10 HU due to macroscopic fat	In the absence of calcification, no further evaluation required for a fat-containing renal lesion < 4 cm in an asymptomatic patient
Suspicious lymph node	Elongated with a central fatty hilum	1 cm cutoff accepted for retroperitoneal nodes
Gastric diverticulum	Fluid-filled or air-filled and usually near the gastric cardia	Often misdiagnosed as a left adrenal mass
Duodenal diverticulum	Pouch with an air-fluid level, typically in the medial wall of the second portion of the duodenum	May be misdiagnosed as a pancreatic mass

In 2002, an interventional radiologist drew upon his own experience as a patient, in order to heat up the discussion about incidental findings^(7)^. Some incidental findings preclude further investigation and, when misdiagnosed, can trigger a false-positive result, which may lead to unnecessary concern on the part of the patients^(1,8)^. In addition, an incidental finding may result in an expensive testing cascade, which can expose the patient to ionizing radiation and, in some cases, invasive procedures, thus increasing morbidity^(7,9)^.

**Figure 1:**
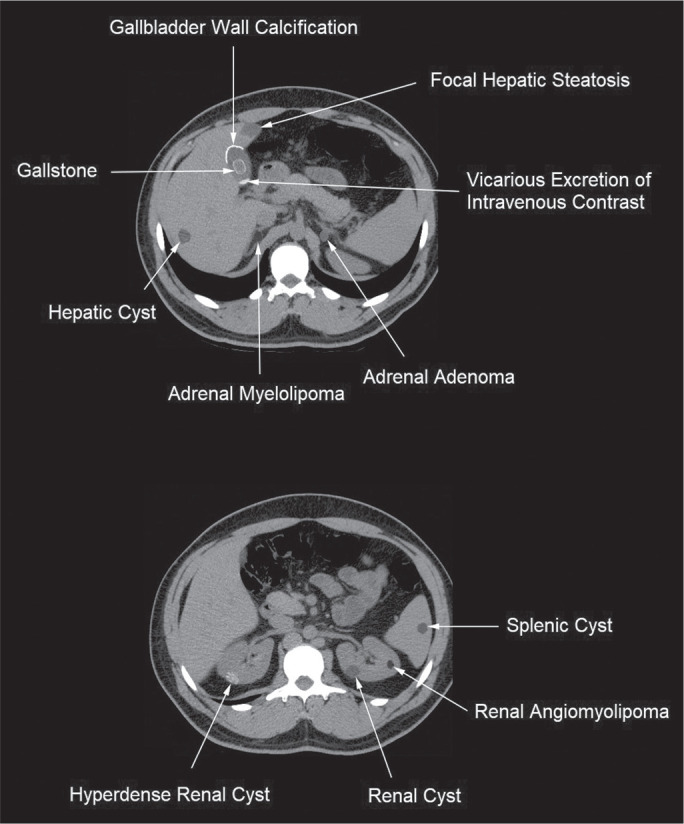
Digitally altered photograph showing common incidental upper abdominal findings that do not require further imaging.

Therefore, the thoracic radiologist must be confident not only in diagnosing clinically insignificant upper abdominal findings but also in reporting when no further investigation is needed, in order to guide the referring physician. The aim of this review article is to summarize the most common incidental upper abdominal findings that do not require further imaging or management in patients undergoing unenhanced CT of the chest for the investigation of thoracic symptoms or diseases. We review common incidental findings of the liver, gallbladder, spleen, adrenal glands, kidney, and retroperitoneum, as well as findings that mimic other lesions (Figure 1).

## LIVER

### Hepatic cyst

**Figure 2:**
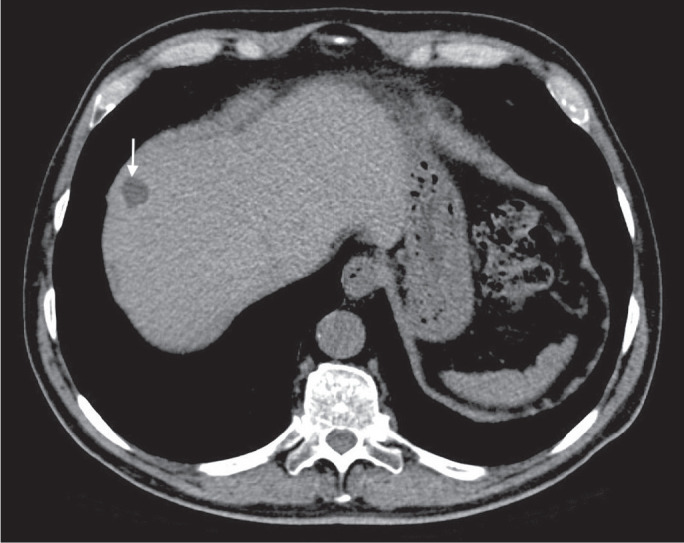
Chest CT of a 44-year-old male, showing a small, well-defined lesion (arrow) with low attenuation (≤ 20 HU), consistent with a hepatic cyst.

A simple hepatic cyst, also known as a bile duct cyst, is a developmental lesion derived from biliary endothelium. One fundamental aspect of such a cyst is that there is no connection between the cyst and the biliary tree^(10)^. This entity is estimated to occur in 2.5% of all people, and its prevalence increases with age^(11)^. The wall of a bile duct cyst is lined by cuboidal epithelium, and the cavity is filled with serous fluid. A simple hepatic cyst is depicted on unenhanced CT as a sharply marginated lesion with well-defined margins, with a density ranging from -10 to 20 HU, and without mural thickening or nodularity^(2,11)^, as illustrated in Figure 2. A simple hepatic cyst detected in an asymptomatic patient with no known malignancy and no hepatic dysfunction does not require further evaluation^(2)^.

### Focal fatty sparing and focal fatty deposition

In a fatty liver, there is accumulation of triglycerides within the cytoplasm of hepatocytes. In that scenario, patients can evolve to nonalcoholic fatty liver disease (NAFLD) or its progressive form, nonalcoholic steatohepatitis (NASH), which presents a risk of cirrhosis, liver cancer, and liver failure^(12)^.

**Figure 3:**
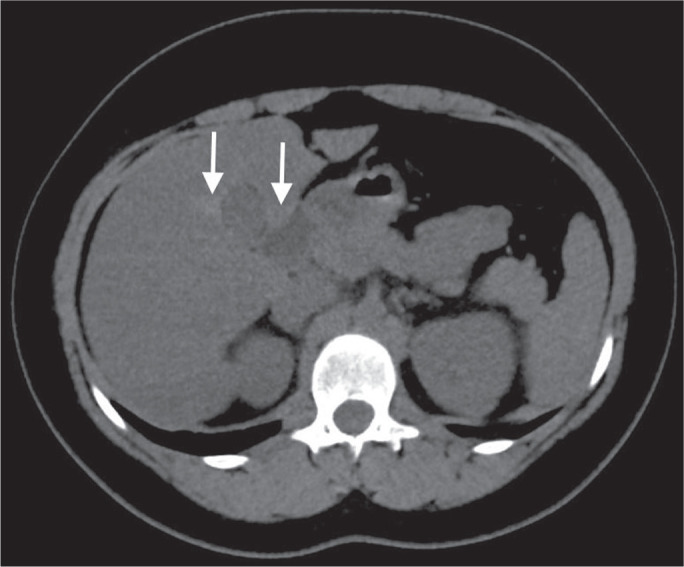
Unenhanced axial CT showing diffuse fat deposition with focal sparing, adjacent to the gallbladder fossa (arrows).

Focal fatty deposition or diffuse fatty deposition with focal sparing (Figure 3) may be misdiagnosed as a focal hepatic lesion. These patterns typically occur adjacent to the falciform ligament, porta hepatis, gallbladder fossa, or subcapsular region^(13)^. One important feature of such deposition is that there is no mass effect on vessels^(14,15)^. On unenhanced CT, there are some criteria proposed to diagnosis fatty liver, such as liver attenuation at least 10 HU less than that of the spleen or less than 40 HU in general^(15)^. In addition, a liver attenuation threshold of 48 HU has been shown to have high specificity for the diagnosis of moderate to severe steatosis^(16)^.

In a retrospective cohort study, Pickhardt et al.^(17)^ found that patients with moderate to severe steatosis did not evolve to NASH. Therefore, isolated incidental steatosis does not necessitate immediate invasive evaluation.

## GALLBLADDER

### Gallstones

Gallstone formation (cholelithiasis) is a common condition that is more prevalent in females, and its prevalence increases with age, regardless of gender^(18,19)^. Other risk factors for cholelithiasis include diabetes mellitus, dyslipidemia, obesity, and rapid weight loss. In a “cohort study of the natural history of gallstones with a long-term follow-up evaluation of a population that was unaware of having gallstones”, Shabanzadeh et al.^(20)^ found that less than 20% of patients underwent cholecystectomy or developed symptoms associated with cholelithiasis, including abdominal pain, acute cholecystitis, common bile duct stones, and pancreatitis.

**Figure 4:**
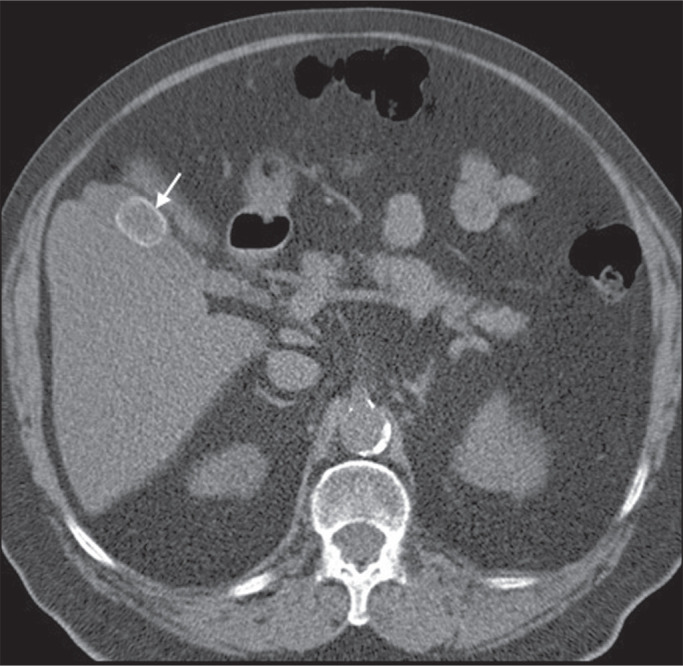
Unenhanced axial CT image showing a gallstone (arrow) in a patient with metastatic angiosarcoma.

Cholesterol is the major component of most gallstones. However, other biochemical structures may constitute a gallstone; calcium bilirubinate is the main constituent of black and brown pigment stones^(21)^. On imaging, CT has a sensitivity of approximately 80% to detect gallstones^(22)^. Therefore, solitary gallstones seen on unenhanced CT (Figure 4) in an asymptomatic patient do not require further imaging^(5)^.

### Porcelain gallbladder

**Figure 5:**
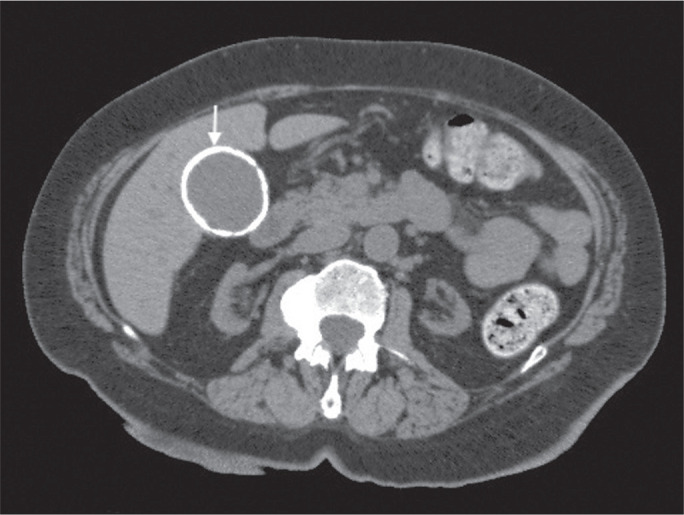
Chest CT of a 66-year-old female with porcelain gallbladder (arrow).

Calcification of the gallbladder wall (Figure 5), also known as porcelain gallbladder, may range from mucosal calcification to complete intramural calcification. This entity predominantly affects women, and the average age at diagnosis is 62 years^(23)^.

Porcelain gallbladder is a risk factor for gallbladder carcinoma. Nevertheless, one meta-analysis found that the incidence of gallbladder carcinoma among patients with porcelain gallbladder is only 6%, which is lower than previously thought^(23)^.

In patients with porcelain gallbladder but no mass, there is no evidence to support imaging follow-up. However, a follow-up imaging examination may be requested, and the decision must be made on a case-by-case basis. If such a patient undergoes follow-up imaging, the recommendation is to use contrast-enhanced CT^(5)^.

### Dense gallbladder content

**Figure 6:**
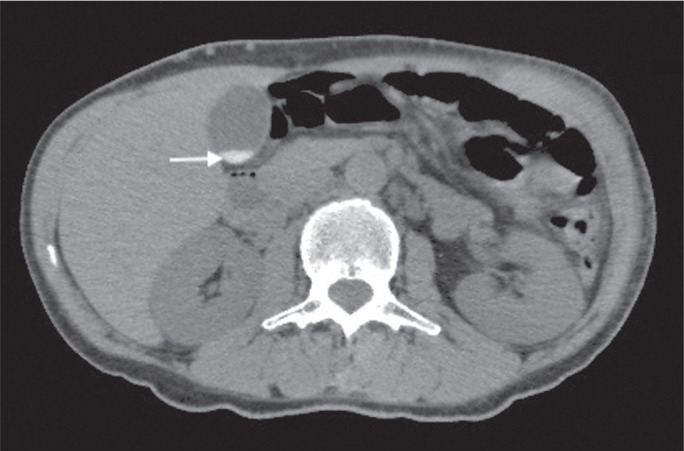
A 52-year-old male with dyspnea. Unenhanced axial CT image showing hyperattenuating gallbladder content (arrow).

There are many causes of gallbladder content that is hyperattenuating, and the clinical history is a valuable tool to narrow the differential diagnosis^(5)^. The liver is an alternative route of the excretion of intravenous (iodinated or gadolinium-based) contrast media, which can result in gallbladder opacification^(5,24)^, as illustrated in Figure 6. The hyperattenuating gallbladder content should raise the suspicion of other possibilities, such as hemorrhage, highly concentrated bile, gallbladder sludge, and noncalcified gallstones. In general, a finding of dense gallbladder content (20-100 HU) on CT with no wall thickening or pericholecystic changes does not require immediate evaluation or follow-up^(5)^.

## SPLEEN

### Splenic cyst

The spleen may not attract the attention of radiologists, probably because it is not necessary for survival^(25)^. However, if the spleen is included in the chest CT scan, a cautious assessment is mandatory.

**Figure 7:**
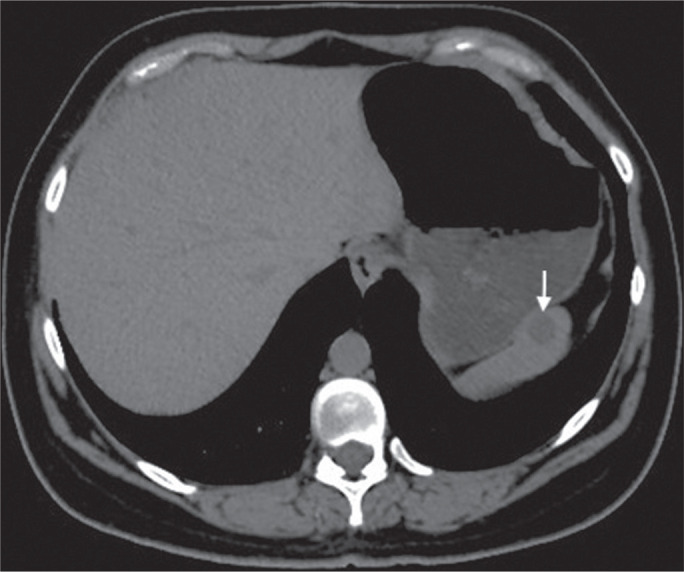
A 36-year-old female. Chest CT, performed for the investigation of cervical lymphadenopathy, showing a splenic cyst (arrow).

One common incidental splenic finding is a cyst, which is a benign lesion diagnosed by its low attenuation (< 10 HU) and the absence of a visible wall^(4)^, as shown in Figure 7. The majority of splenic cysts are secondary (false) cysts, rather than primary (true) cysts. An epithelial lining characterizes true cysts, which are typically congenital, whereas false cysts have a fibrous wall, and their cystic nature is due to liquefactive necrosis caused by a previous trauma, infection, or infarction. On CT, true and false cysts are indistinguishable. In areas where hydatid disease is endemic, a parasitic cyst should be considered. Although some metastases may be cystic, isolated splenic metastasis is uncommon^(4,25,26)^.

## ADRENAL GLANDS

### Lipid-rich adenoma

The most common adrenal lesion is a cortical adenoma^(27)^. In most autopsy studies, the prevalence of adrenal cortical adenoma ranges from 1.38% to 8.9%^(28)^. Such adenomas are usually nonfunctioning^(29)^. In a pooled analysis of ten studies evaluating the accuracy of unenhanced CT to discriminate between benign and malignant adrenal lesions, conducted in 1998, Boland et al.^(30)^ found that an attenuation cutoff of ≤ 10 HU has a sensitivity and specificity of 71% and 98%, respectively, for the diagnosis of a benign adrenal lesion. Their analysis comprised 495 adrenal lesions, of which 275 were benign. Of the benign lesions, 261 were adenomas.

**Figure 8:**
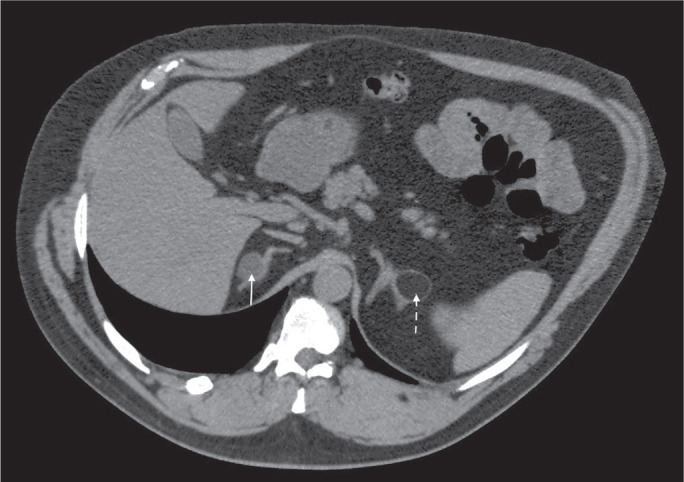
A 54-year-old male patient with Crohn’s disease. Axial oblique CT showing an adenoma (solid arrow) in the right adrenal gland and a myelolipoma (dashed arrow) in the left adrenal gland.

Adenomas are usually characterized as lipid-rich and lipid-poor. Unenhanced CT can identify lipid-rich adenomas because they contain abundant intracellular fat, resulting in an attenuation value ≤ 10 HU. However, attenuation values > 10 HU on unenhanced CT may represent not only lipid-poor adenoma but also non-adenomatous lesions, including metastasis and pheochromocytoma^(29,31)^. Lipid-rich adenomas account for up to 70% of adenomas, and an adrenal mass with a density ≤ 10 HU on unenhanced CT is indicative of lipid-rich adenoma (Figure 8), regardless of size^(1,29)^.

### Myelolipoma

**Figure 9:**
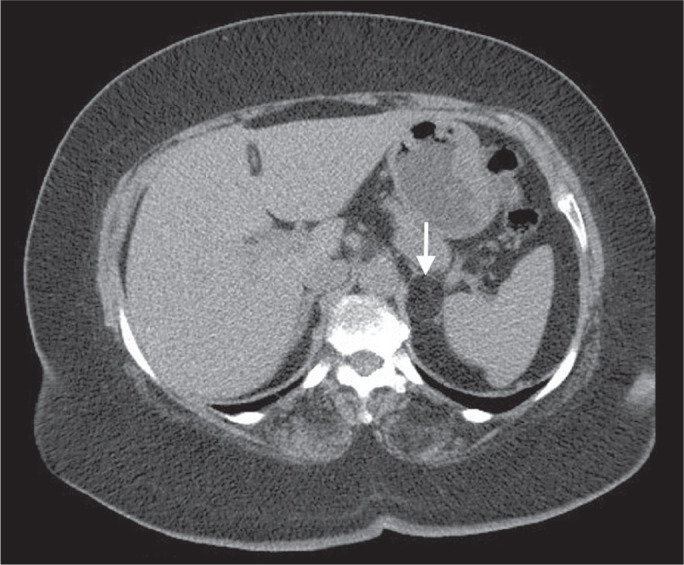
Adrenal myelolipoma in a 60-year-old woman. Unenhanced axial CT image showing a mass containing macroscopic fat (arrow) in the left adrenal gland.

The main components of an adrenal myelolipoma are adipose tissue and hematopoietic elements, and 24% of isolated adrenal myelolipomas present calcification^(27,32,33)^. Myelolipomas are typically identified incidentally and account for 6% of all adrenal lesions detected on CT in patients with no history of cancer^(34)^. Although most myelolipomas are asymptomatic, symptoms may develop, especially in larger lesions either due to a mass effect or internal hemorrhage^(33)^. Macroscopic fat is the main diagnostic feature on unenhanced CT (Figures 8 and 9), and no additional imaging is needed^(1)^.

## KIDNEYS

### Benign cyst

**Figure 10:**
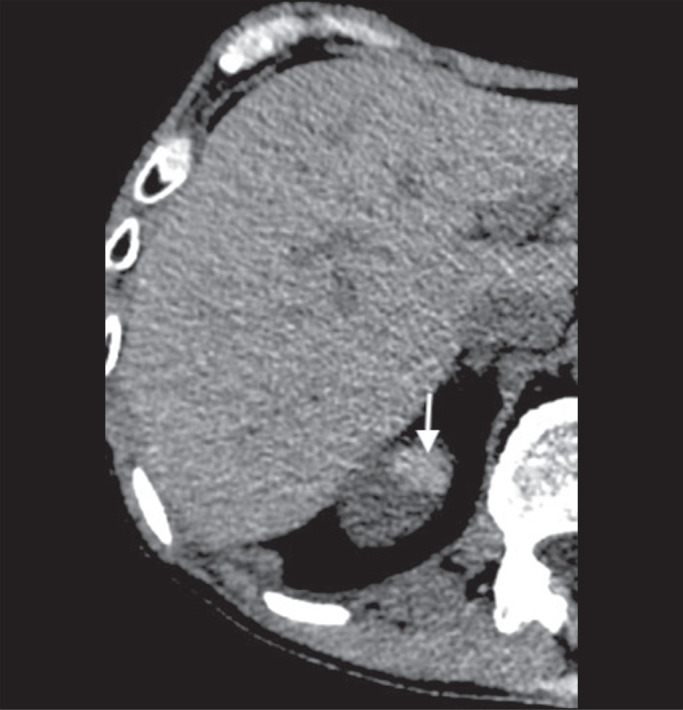
A 67-year-old male with dyspnea. Chest CT showing a hyperdense renal cyst (arrow).

Most cystic renal masses are benign, and it is estimated that they occur in 41% of patients undergoing abdominal CT for an unrelated reason^(35)^. In 1986, the Bosniak renal cyst classification system was introduced^(36)^. The system divides such masses into five categories (I, II, IIF, III, and IV), according to their morphology and enhancement characteristics^(37)^. Although the Bosniak classification does not incorporate incompletely characterized masses, a recent proposal is that masses that are highly likely to be benign should be classified as Bosniak II masses. On unenhanced CT, well-defined homogeneous masses with a density ranging from -9 to 20 HU or ≥ 70 HU (Figure 10) are highly likely to be benign, therefore requiring no follow-up^(3,38-40)^. High attenuation of a renal cyst may be due to hemorrhage or high protein content.

### Angiomyolipoma

Angiomyolipoma (AML) is a common mesenchymal tumor of the kidney, typically composed of fat, blood vessels, and smooth muscle, in varying proportions. Most AMLs are sporadic; they are usually solitary and predominantly affect women, at a female:male ratio of 4:1. However, when they occur in patients with tuberous sclerosis, renal AMLs are commonly multiple, with no sex predilection^(41,42)^. Although most AMLs are asymptomatic, patients with larger lesions are more likely to present a palpable mass, flank pain, and hematuria^(41)^. Tumor size ≥ 4 cm and an aneurysm > 5 mm within the tumor are predictors of rupture, the latter having higher specificity^(43)^.

**Figure 11:**
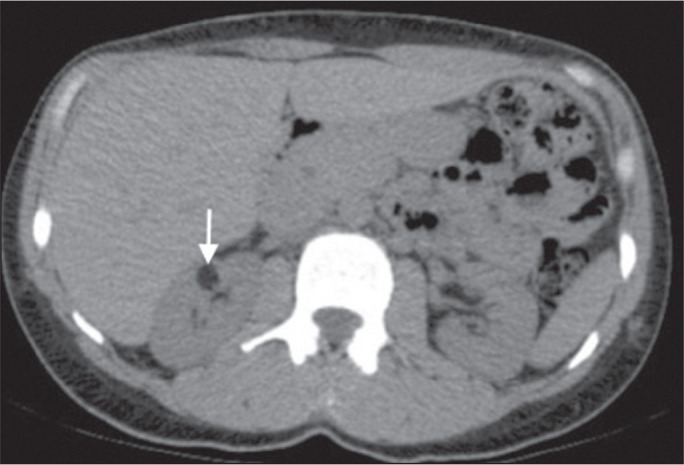
A 39-year-old female with dyspnea. Chest CT showing a very low-density lesion (arrow) in the right kidney.

A classification system proposed by Song et al.^(44)^ categorizes AMLs as fat-rich, fat-poor, or fat-invisible, based on the quantity of fat identified on unenhanced CT or magnetic resonance imaging. Fat-poor and fat-invisible AMLs cannot be classified solely with unenhanced CT, and their differential diagnoses include renal cell carcinoma. In contrast, fat-rich AMLs, which are the most common AMLs, can be identified on unenhanced CT by a density ≤ -10 HU due to macroscopic fat^(44)^, as depicted in Figure 11. Calcifications are rare in AMLs. Although uncommon, macroscopic fat can be seen in a renal cell carcinoma, and calcifications within the tumor are more common in such cases^(45)^. Therefore, in the absence of calcification, a fat-containing renal lesion < 4 cm in an asymptomatic patient does not require further evaluation^(3)^.

## LYMPH NODES

When detected as incidental findings, the majority of abnormal abdominal lymph nodes are benign; if they meet certain criteria, no further investigation is needed^(4)^. The transverse diameter should be assessed on the short axis, rather than on the long axis. The variability in the diameter of a node, which depends on its spatial orientation, is less pronounced in short-axis measurements^(46)^. Although a 1 cm cutoff is accepted to discriminate between normal and suspicious lymph nodes in the retroperitoneum, there is little evidence to support its use in other contexts. Therefore, features other than size are used in order to determine whether a lymph node is benign or suspicious; for example, a reniform shape with a central fatty hilum is indicative of a benign lymph node^(4)^.

## MIMICS

### Gastric diverticulum

**Figure 12:**
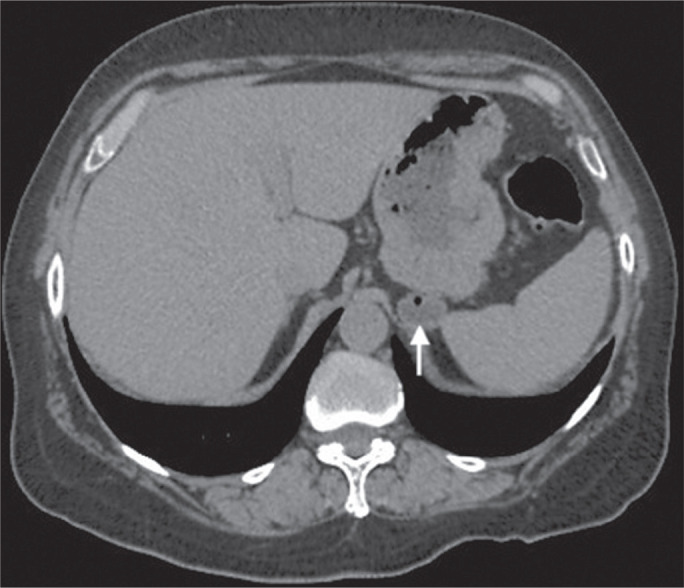
A 65-year-old female with rheumatoid arthritis. Chest CT showing a gastric diverticulum (arrow).

Gastric diverticulum is an uncommon abnormality, and it may be congenital or acquired. Among the types gastric diverticula, acquired diverticulum is the least common. Congenital diverticulum, also known as true gastric diverticulum, includes all stomach wall layers. The majority of true gastric diverticula are in the cardia region of the stomach, on the posterior aspect of the lesser curvature. Gastric diverticulum is often misdiagnosed as a left adrenal mass. Unenhanced CT may show a fluid-filled or an air-filled pouch (Figure 12), and the communication with the gastrointestinal tract may not be obvious^(47,48)^.

### Duodenal diverticulum

**Figure 13:**
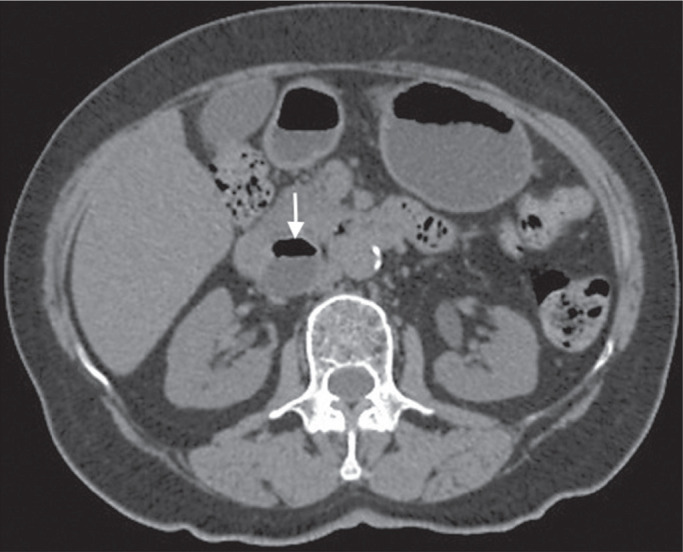
An 83-year-old female. Chest CT showing a duodenal diverticulum (arrow) discovered as an incidental finding after blunt chest trauma.

The duodenum is a common site of diverticula in the digestive tract. In many cases, the mucosa and muscularis mucosa layers herniate through the medial wall of the second portion of the duodenum, probably due to weak spots caused by penetrating vessels. The third and fourth portions of the duodenum are less affected. Most patients with duodenal diverticula do not develop symptoms, although diverticulitis, perforation, and hemorrhage may occur. Albeit uncommon, a duodenal diverticulum may compress the common bile duct, resulting in obstruction and jaundice (Lemmel’s syndrome). On unenhanced CT, a duodenal diverticulum appears as a pouch with an air-fluid level (Figure 13), occasionally mimicking a pancreatic mass^(49,50)^.

## CONCLUSION

Thoracic radiologists should be aware of the characteristics of incidental findings in the upper abdomen, in order to guide the referring physicians. In addition, the thoracic radiologist plays a crucial part in patient care, given that a reliable diagnosis of a benign lesion protects patients from additional medical interventions and allays patient concerns.
